# Supplementary material for: Evolving copy number gains promote tumor expansion and bolster mutational diversification
Source: Nat Commun. 2024 Mar 6;15:2025. doi: 10.1038/s41467-024-46414-5 (PMC10918155; doi:10.1038/s41467-024-46414-5)
Supplement: Supplementary file 1 — Supplementary Information [file 41467_2024_46414_MOESM1_ESM.pdf]

Supplementary information: Evolving copy number gains promote tumor expansion and bolster mutational diversification

Zicheng Wang[1,2,3†], Yunong Xia[1,2], Lauren Mills[4], Athanasios N. Nikolakopoulos[1,2], Nicole Maeser[1,2], Scott M. Dehm[1,2,5], Jason M. Sheltzer[6], Ruping Sun[1,2*†]

[1*]Department of Laboratory Medicine and Pathology, University of Minnesota, Minneapolis, Minnesota, United States of America.
[2]Masonic Cancer Center, University of Minnesota, Minneapolis, Minnesota, United States of America.
[3]School of Data Science, The Chinese University of Hong Kong (CUHK-Shenzhen), Shenzhen, China.
[4]Department of Pediatrics, University of Minnesota, Minneapolis, Minnesota, United States of America.
[5]Department of Urology, University of Minnesota, Minneapolis, Minnesota, United States of America.
[6]School of Medicine, Yale University, New Haven, Connecticut, United States of America.

*Corresponding author(s). E-mail(s): ruping@umn.edu;
†These authors contributed equally to this work.

Supplementary Methods

Mathematical modeling of the late evolving gains

In the first model, we aim to obtain the number of mutations which are shared by more than 90% of the total population. We utilize the following result from [1], which gives a formula for the number of mutations that are found in a certain number of skeleton cells (skeleton cells are those with an infinite line

of descent). Define

$$\tau_N := \inf\{t \geq 0 : Z_0(t) = N\},$$

as the random time at which the skeleton subpopulation reaches size N at the first time. Let $S_j(t)$ denote the number of mutations that are found in $j \geq 1$ skeleton cells at time t conditioned on the non-extinction of the cancer cell population. Equation C.1 of [1] states that

$$\mathbb{E}\left[S_j(\tau_N)\right] = \begin{cases} \frac{u_0}{\lambda_0} N \cdot 1/\left(j(j+1)\right) - \frac{u_0}{\lambda_0}\delta_{1,j}, & 1 \leq j \leq N-1 \\ \frac{u_0}{\lambda_0}, & j = N, \end{cases}$$

where $\delta_{l,m} = 1$ if $l = m$ and $\delta_{l,m} = 0$ otherwise. Therefore, we can obtain that

$$\begin{aligned} \tilde{S} &= \sum_{j=\lceil 0.9N \rceil}^{N} \mathbb{E}\left[S_j(\tau_N)\right] \\ &= \frac{u_0}{\lambda_0} + \sum_{j=\lceil 0.9N \rceil}^{N-1} \frac{u_0}{\lambda_0} N \cdot 1/\left(j(j+1)\right) \\ &= \frac{u_0}{\lambda_0} + \frac{u_0}{\lambda_0} N \left(\frac{1}{\lceil 0.9N \rceil} - \frac{1}{N}\right) \\ &= \frac{N}{\lceil 0.9N \rceil} \cdot \frac{u_0}{\lambda_0} \\ &\approx 1.11 \frac{u_0}{\lambda_0}. \end{aligned}$$

In the second model, we obtained the expected number of passenger mutations accumulated in the first type 1 cell with infinite lineage, denoted by $\bar{S}$:

$$\bar{S} = \int_0^\infty \mathbb{P}\left(\sigma_1 > t \mid \Omega_\infty\right) u_0 dt. \tag{1}$$

It remains to prove Lemma 1. Time until the first type 1 cell has been studied in existing literature with a deterministic approximation of the evolution of type 0 cells (see [2] and references therein). In Lemma 1, we obtained the exact distribution.

Lemma 1. Let σ_1 denote the time of occurrence of the first type 1 cell that gives rise to a family which does not die out, and let Ω_∞ denote the event of non-extinction of the tumor. Then

$$\mathbb{P}\left(\sigma_1 > t \mid \Omega_\infty\right) = \frac{a_0(1-q_0) + \frac{u_1(1-q_1)}{1-q_0}}{a_0(1-q_0) + \frac{u_1(1-q_1)}{1-q_0} e^{\zeta t}},$$

where

$$
\begin{aligned}
q_0 &= \frac{a_0 + b_0 + u_1 - \sqrt{\left(a_0 + b_0 + u_1\right)^2 - 4a_0\left(u_1 q_1 + b_0\right)}}{2a_0}, \\
q_1 &= \frac{b_1}{a_1}, \quad \text{and} \\
\zeta &= \frac{u_1\left(1 - q_1\right)}{1 - q_0} + a_0\left(1 - q_0\right).
\end{aligned}
$$

Proof. Recall that type 0 cells give birth at a rate of a_0 and die at a rate of b_0. The net growth rate is $\lambda_0 = a_0 - b_0 > 0$. Type 0 cells also mutate to type 1 cells at rate u_1. Type 1 cells give birth at a rate of a_1 and die at a rate of b_1. The net growth rate is $\lambda_1 = a_1 - b_1 > \lambda_0$.

Let q_0 denote the extinction probability of a type 0 cell, and q_1 the extinction probability of a type 1 cell. By considering what happened on the first event of a type 0 and a type 1 cell, we have

$$
\begin{aligned}
q_0 &= \frac{b_0}{a_0 + b_0 + u_1} + \frac{a_0}{a_0 + b_0 + u_1} q_0^2 + \frac{u_1}{a_0 + b_0 + u_1} q_1 \\
q_1 &= \frac{b_1}{a_1 + b_1} + \frac{a_1}{a_1 + b_1} q_1^2.
\end{aligned}
$$

We can then obtain that

$$
\begin{aligned}
q_0 &= \frac{a_0 + b_0 + u_1 - \sqrt{\left(a_0 + b_0 + u_1\right)^2 - 4a_0(u_1 q_1 + b_0)}}{2a_0} \\
q_1 &= \frac{b_1}{a_1}.
\end{aligned}
$$

To obtain the infinitesimal generator for the skeleton subpopulation process of type 0 and type 1 cells conditioned on the event that the population (starting from a type 0 cell) does not go extinct, we analyze its discrete time approximations and take a limit (see [3] or Section 3.1 of [2]). We can then obtain the infinitesimal generator for the skeleton process:

$$
\begin{bmatrix} a_0\left(1 - q_0\right) & \frac{u_1(1-q_1)}{1-q_0} \\ 0 & a_1 - b_1 \end{bmatrix},
$$

where $a_0\left(1 - q_0\right)$ is the birth rate of a type 0 cell, $a_1 - b_1$ is the birth rate of a type 1 cell, and $\frac{u_1(1-q_1)}{1-q_0}$ is the rate of switching from a type 0 to a type 1 cell.

For simplicity, let $f(t) = \mathbb{P}\left(\sigma_1 > t \mid \Omega_\infty\right)$. We then apply Theorem 8 in [4] and obtain the following differential equation for $f(t)$:

$$
\frac{df\left(t\right)}{dt} = a_0\left(1 - q_0\right) f\left(t\right)^2 - \zeta f\left(t\right),
$$

where

$$\zeta = \frac{u_1 (1 - q_1)}{1 - q_0} + a_0 (1 - q_0).$$

Lastly, we solve the differential equation and obtain that

$$\mathbb{P}(\sigma_1 > t \mid \Omega_\infty) = \frac{a_0 (1 - q_0) + \frac{u_1 (1 - q_1)}{1 - q_0}}{a_0 (1 - q_0) + \frac{u_1 (1 - q_1)}{1 - q_0} e^{\zeta t}}.$$

Supplementary Tables

Notation and Terminology	Definition
SCNA	Somatic copy number aberration
SSNV	Somatic single nucleotide variant
CN	Copy Number
SV	Structural variant
SFS	Site frequency spectrum of SSNVs
Evolution time	Truncal evolution time measured by clonal SSNVs from tumor samples
Nt	Total copy number of a DNA segment
Nb	Minor copy number of a DNA segment
MRCA	Most recent common ancestor of the tumor sample(s)
Initiation Time (t_0)	Time fraction from germline to the first gain
Lead Time (t_K)	Time fraction from the establishment of the observed copy number state to the MRCA
Arrival Time	Time fraction from germline to the establishment of the observed copy number state (i.e., $1 - t_K$)
SRE	Sampling-relevant expansion, i.e., the population expansion that leads to the observed tumor sample, which may only involve subclone(s) of the entire tumor.
Final-expansion driver	Driver mutation that is responsible for the fitness increase leading to the final expansion of a tumor
GD	The prominent and concentrated burst of gains, containing more than 40% of all timed genomic segments.
Early gains	Gains occurring in the first 20% of the truncal evolution time measured by clonal SSNVs.
Late gains	Gains occurring in the last 20% of the truncal evolution time measured by clonal SSNVs.
a_0	Birth rate of cancer cells with no post-GD beneficial gains
b_0	Death rate of cancer cells with no post-GD beneficial gains
λ_0	Growth rate of cancer cells with no post-GD beneficial gains
a_1	Birth rate of cancer cells with a post-GD beneficial gain
b_1	Death rate of cancer cells with a post-GD beneficial gain
λ_1	Growth rate of cancer cells with a post-GD beneficial gain
u_0	Mutation rate of passenger gain
u_1	Mutation rate of post-GD beneficial gain

Supplementary Table S 1 Table of Notation and Terminology.

Supplementary Figures

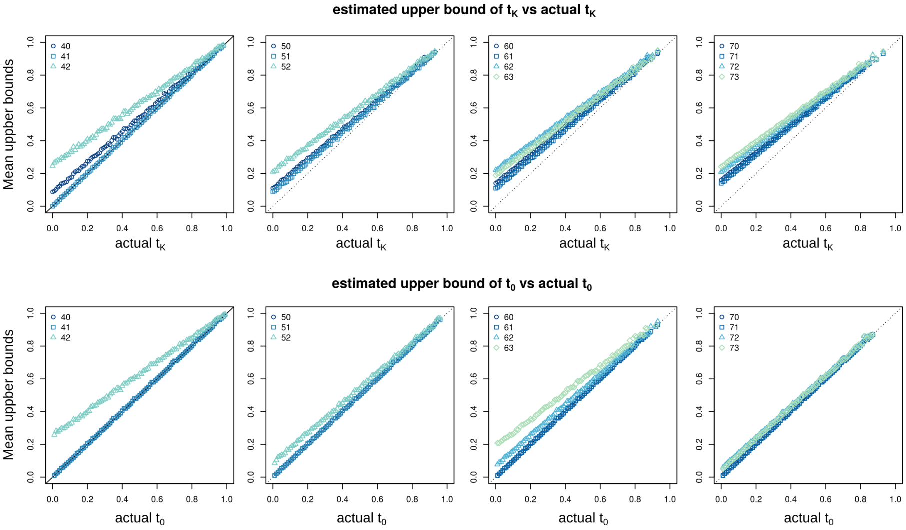

Supplementary Figure S 1 Estimated timing systematically correlates with the actual timing. The upper panel shows the mean lead time (upper bounds of t_K) against the actual t_K in simulations. The lower panel demonstrates the initiation time. Each plot is for a specific total CN state, where point shapes and colors indicate the complete SCNA configuration (with the format of total CN:minor CN, or $Nt : Nb$). We simulated 5000 timing combinations for each history matrix in a specific SCNA configuration. In these simulations, the true allele state distribution was used and we neglected the noise contained in the actual sequencing data. Simulations are binned based on the actual timing of t_K and t_0 (within 0.01 interval), respectively. Each point refers to the averaged timing estimate for each bin. Source data are provided as a Source Data file.

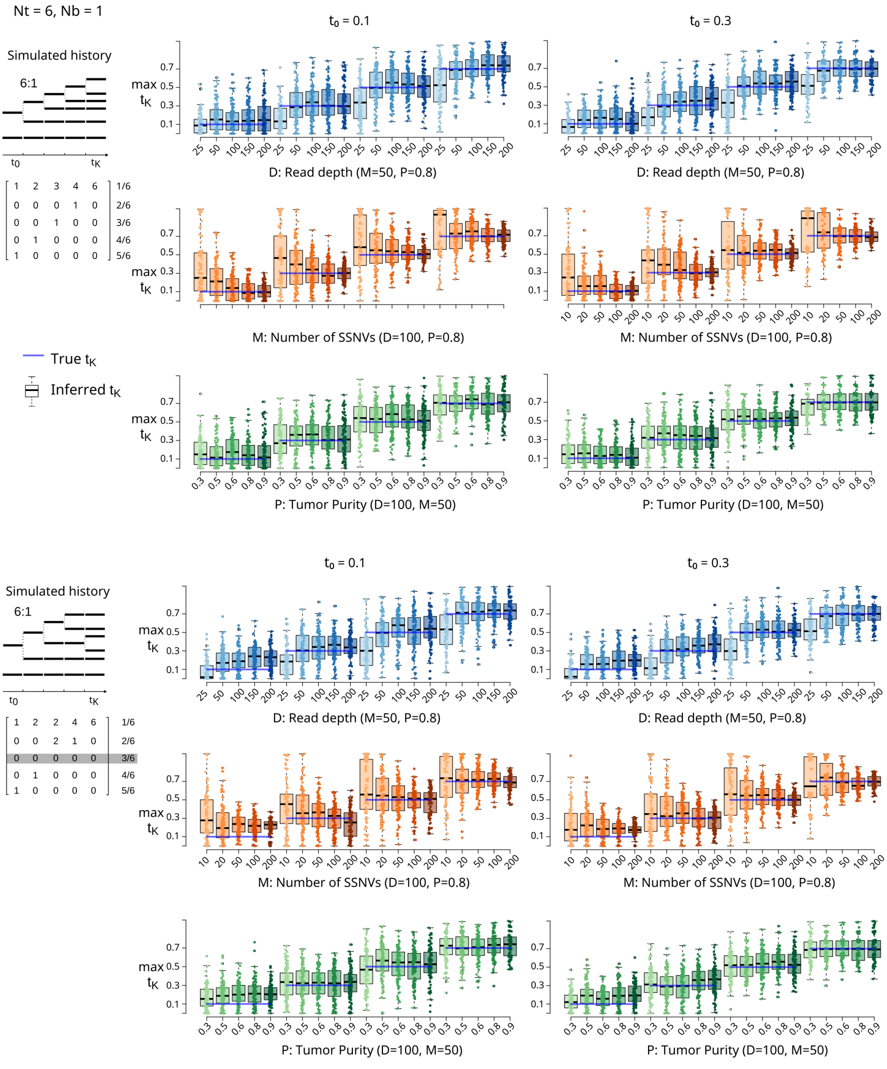

Supplementary Figure S 2 The performance of `Butte` on estimating t_K for SCNA state 6:1 with simulated history and SSNV data. We simulated SSNV data for the SCNA state 6:1 for two different histories (shown on the left panel), with varying t_K (0.1, 0.3, 0.5, 0.7) for two pre-defined t_0 (0.1 and 0.3). The intermediate time stages between t_0 and t_K were equally split. For a given history, we simulated SSNV data with varying depth of coverage (D), number of available mutations (M) and tumor purity (P). To replicate the depth filter in variant calling, we filtered out simulated SSNVs lacking adequate alternative read counts (less than 3). The inferred upper bound of t_K by `Butte` are shown as standard box plots (each with 100 simulations), and the true t_K are indicated by blue lines. The box represents the interquartile range, covering the central 50% of the data. The line inside the box indicates the median. Whiskers extend to the minimum and maximum values within a specified range, excluding outliers. Source data are provided as a Source Data file.

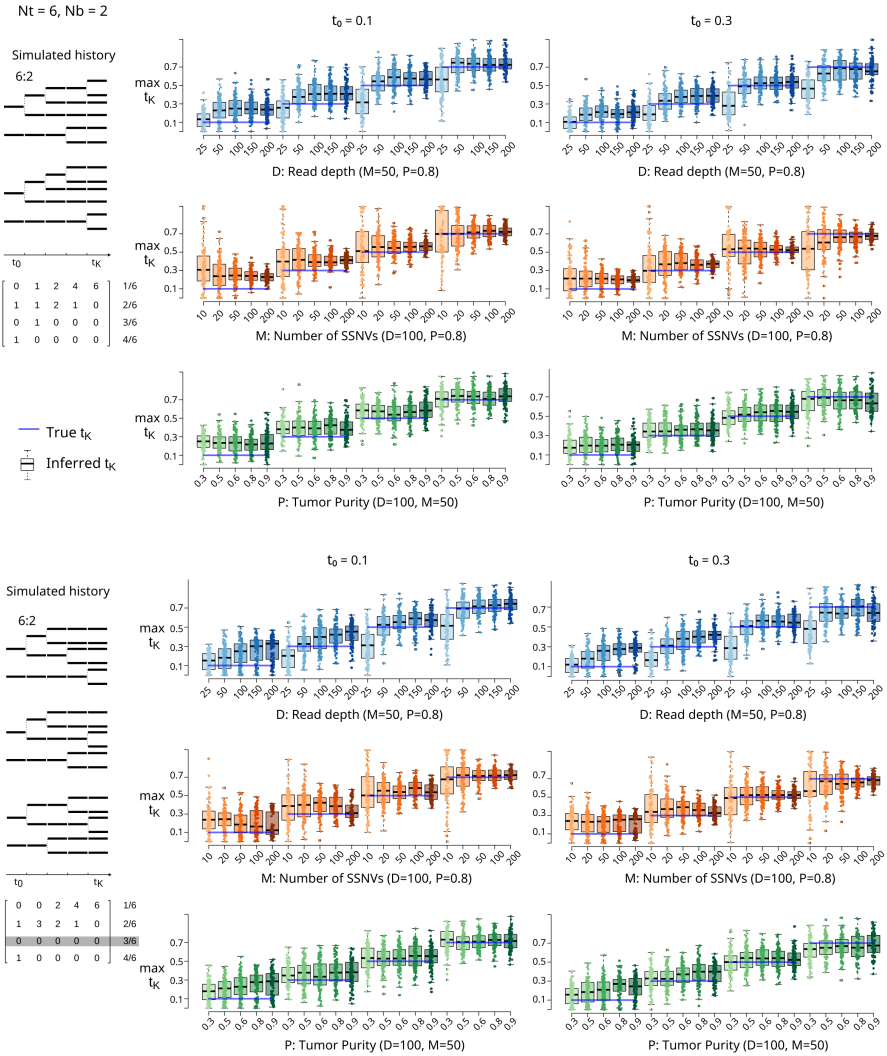

Supplementary Figure S 3 The performance of `Butte` on estimating t_K for SCNA state 6:2 with simulated history and SSNV data. The plots are arranged similarly as in Figure S2. The inferred upper bound of t_K by `Butte` are shown as standard box plots (each with 100 simulations), and the true t_K are indicated by blue lines. The box represents the interquartile range, covering the central 50% of the data. The line inside the box indicates the median. Whiskers extend to the minimum and maximum values within a specified range, excluding outliers. To replicate the depth filter in variant calling, we filtered out simulated SSNVs lacking adequate alternative read counts (less than 3). Note that as we do not distinguish the SSNVs between maternal and paternal alleles, the same history matrix (and the resulting site frequency spectrum of SSNVs) can represent different gain trajectories on the two alleles. Source data are provided as a Source Data file.

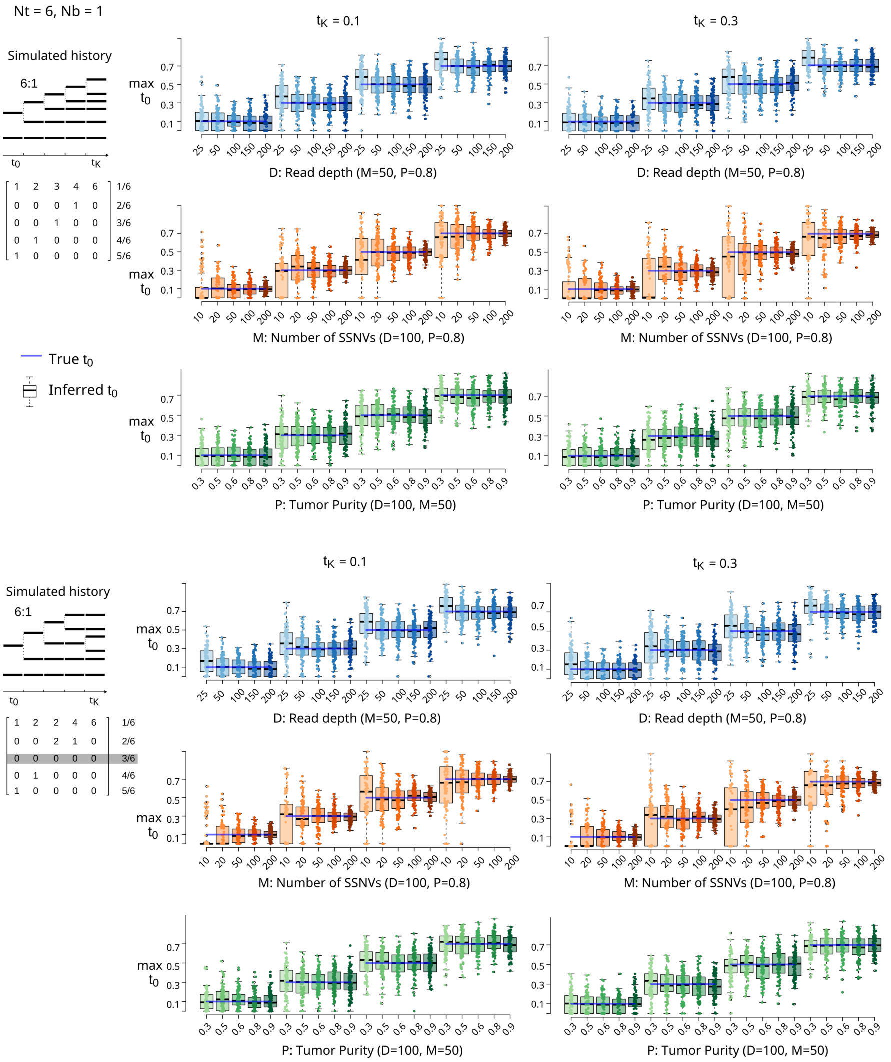

Supplementary Figure S 4 The performance of `Butte` on estimating t_0 for SCNA state 6:1 with simulated history and SSNV data. We simulated SSNV data for the SCNA state 6:1 for two different histories (shown on the left panel), with varying t_0 (0.1, 0.3, 0.5, 0.7) for two pre-defined t_K (0.1 and 0.3). The intermediate time stages between t_0 and t_K were equally split. For a given history, we simulated SSNV data with varying depth of coverage (D), number of available mutations (M) and tumor purity (P). To replicate the depth filter in variant calling, we filtered out simulated SSNVs lacking adequate alternative read counts (less than 3). The inferred upper bound of t_0 by `Butte` are shown as standard box plots (each with 100 simulations), and the true t_0 are indicated by blue lines. The box represents the interquartile range, covering the central 50% of the data. The line inside the box indicates the median. Whiskers extend to the minimum and maximum values within a specified range, excluding outliers. Source data are provided as a Source Data file.

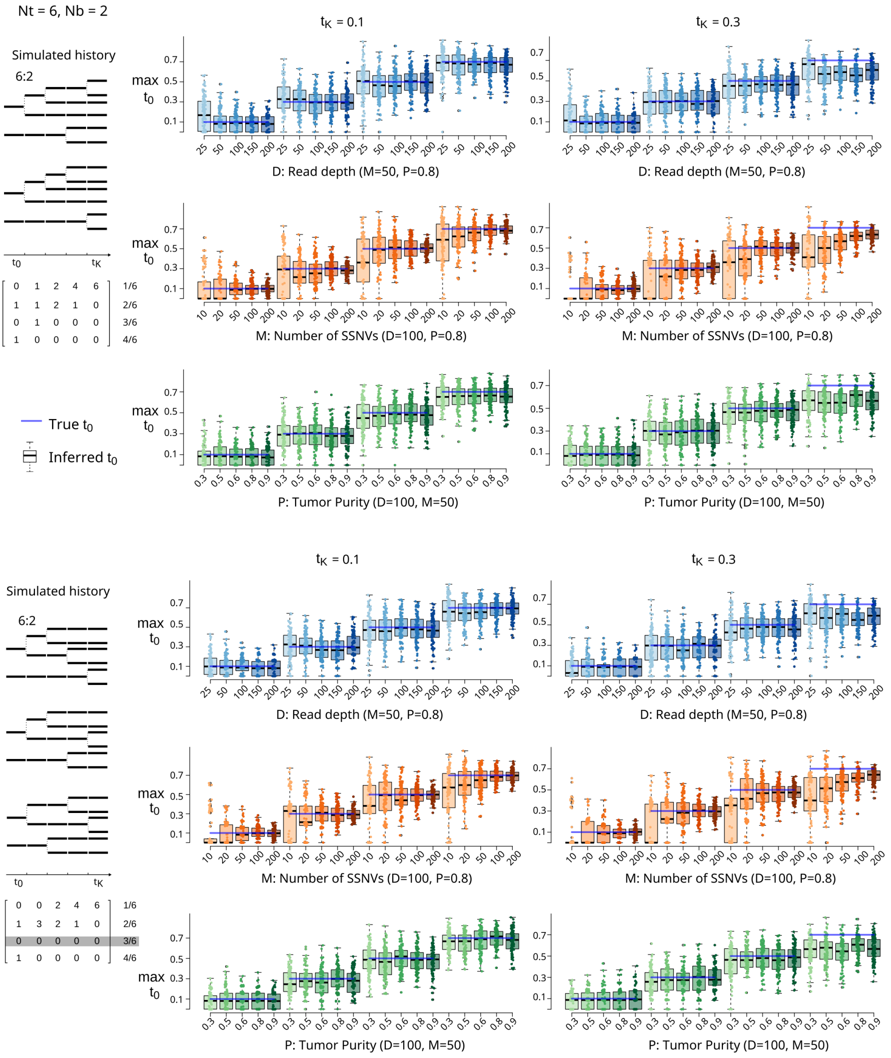

Supplementary Figure S 5 The performance of `Butte` on estimating t_0 for SCNA state 6:2 with simulated history and SSNV data. The plots are arranged similarly as in Figure S4. The inferred upper bound of t_0 by `Butte` are shown as standard box plots (each with 100 simulations), and the true t_0 are indicated by blue lines. The box represents the interquartile range, covering the central 50% of the data. The line inside the box indicates the median. Whiskers extend to the minimum and maximum values within a specified range, excluding outliers. To replicate the depth filter in variant calling, we filtered out simulated SSNVs lacking adequate alternative read counts (less than 3). Note that as we do not distinguish the SSNVs between maternal and paternal alleles, the same history matrix (and the resulting site frequency spectrum of SSNVs) can represent different gain trajectories on the two alleles. Source data are provided as a Source Data file.

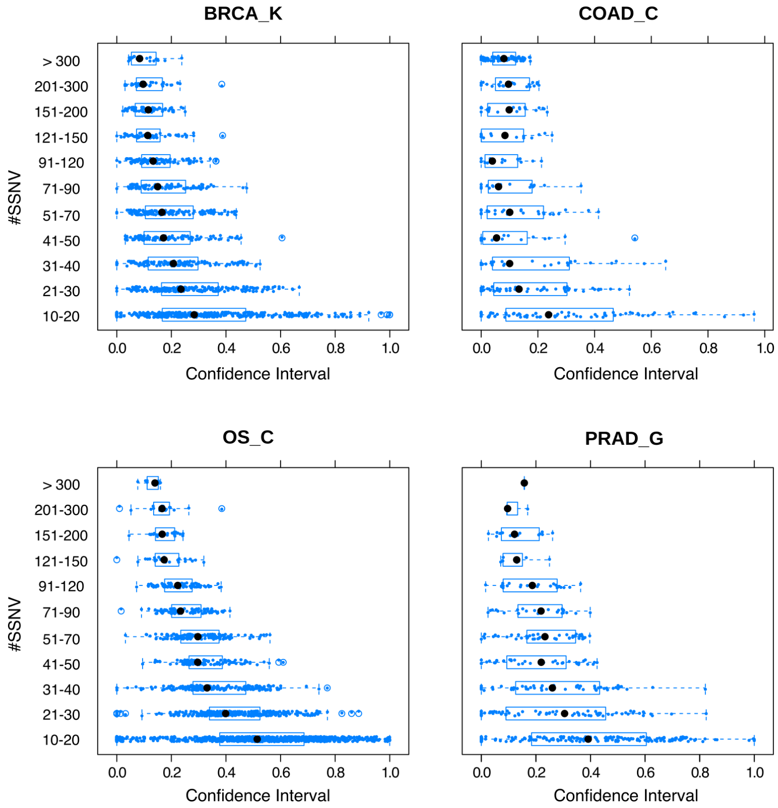

Supplementary Figure S 6 Box plots categorize the distribution of 90% confidence intervals for estimated t_K across four datasets based on the available number of SSNVs for estimation. Larger numbers of SSNVs result in smaller confidence intervals, indicating increased precision in the estimates. The box represents the interquartile range, covering the central 50% of the data. The line inside the box indicates the median. Whiskers extend to the minimum and maximum values within a specified range, excluding outliers. Source data are provided as a Source Data file.

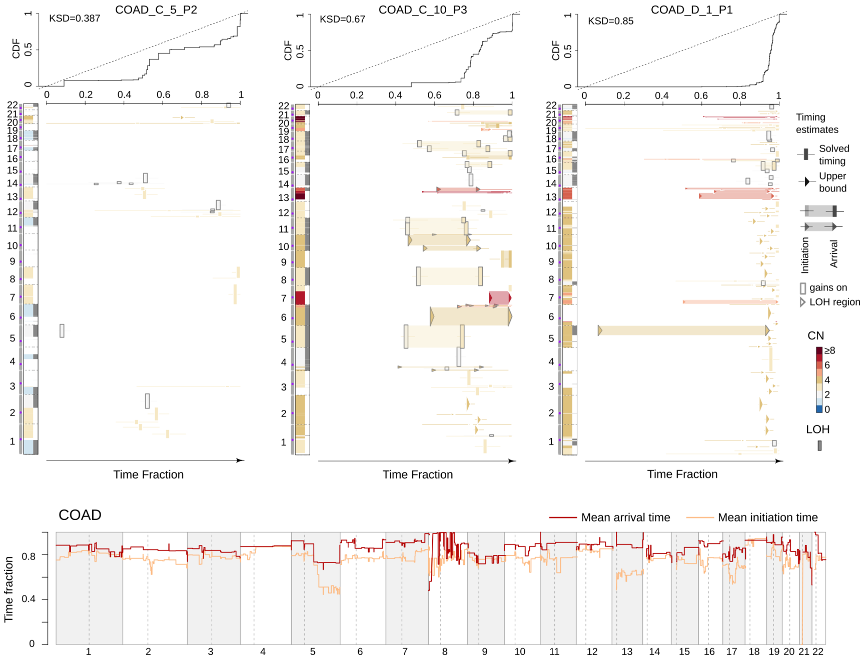

Supplementary Figure S 7 The timing patterns of SCNAs in COAD. The upper panel illustrates the SCNA evolution of three exemplified COAD tumors. CN states along the genome are shown on the left of each panel. The right panel visualizes the time fraction of somatic evolution from germline to the MRCA of the patient tumor. For each SCNA segment, the inferred timing is drawn by either rectangle (exactly solved timing) or arrows (upper bounds of timing when the solutions are not unique) with the same color-coding as its CN. The top panel shows the cumulative distribution (CDF) of SCNA arrival time. The CDF curve of SCNA arrival time is shown for each patient categorised by the tumor type. In the lower panel, the average timing (both initiation and arrival time for each one Megabase genomic bin across COAD patients are shown, respectively. Source data are provided as a Source Data file.

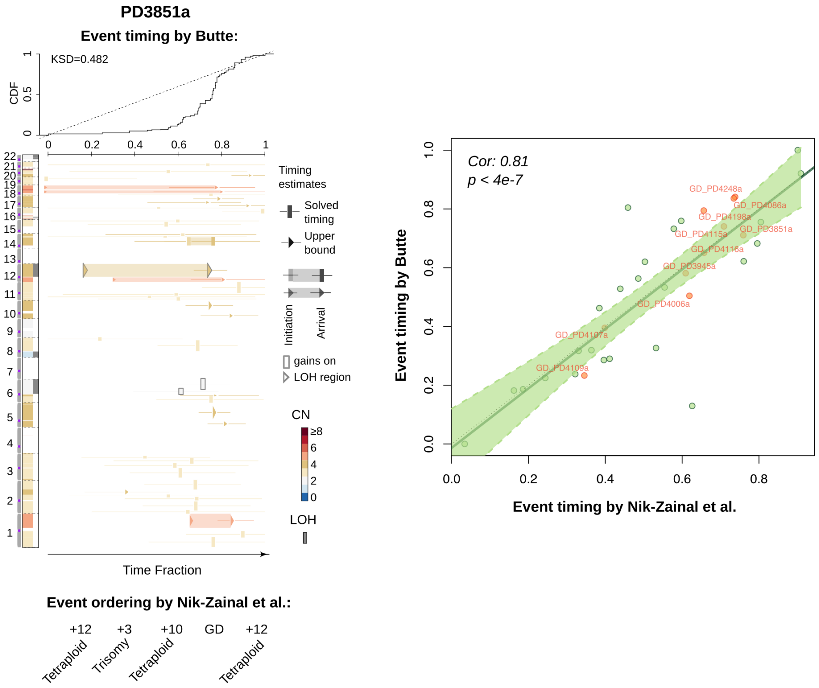

Supplementary Figure S 8 Butte's predictions for SCNAs, encompassing tetraploidy, trisomy, tandem duplication, and GD, are in agreement with those documented by [5] through the graph theory-based method [6]. To analyze tumors from [5] with Butte, we obtained SSNV and SCNA predictions for the same samples from the PCAWG (PanCancer Analysis of Whole Genomes) dataset [7] via the ICGC data portal. The left panel displays an illustrative case (PD3851a) with event sequencing as reported by [5] below Butte's findings. In the right panel, a scatter plot compares the timing predictions of the two methods across 15 patients, with GD events highlighted in red dots. The Spearman correlation coefficient and corresponding p-value are presented. It's important to note that the comparison includes only events reported by [5], although Butte is capable of predicting timing for additional events. Source data are provided as a Source Data file.

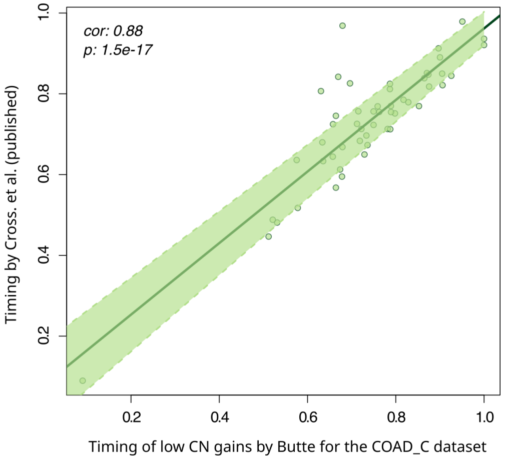

Supplementary Figure S 9 The timing of single and double gains (with the SCNA setting at 2:0, 3:1 and 4:2) reported by [8] is plotted against the inferred timing by `Butte`. As there could be differences in the SCNA segmentation between Cross' method and ours, for each SCNA segment timed by Cross et. al., we located the segment with the matching chromosome, copy number state and segment length. We then extracted the timing inferred by `Butte`. Overall our timing for these low CN gains agrees with Cross' method. Source data are provided as a Source Data file.

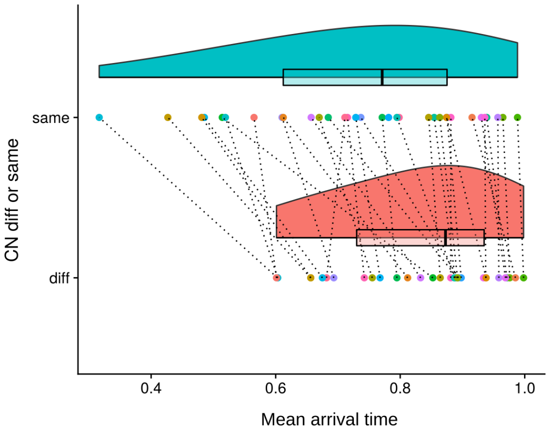

Supplementary Figure S 10 We conducted pairwise sample comparisons in each patient with multiple sampling, encompassing COAD, BRCA, PRAD, ESCA, and OS datasets, to identify genomic regions with stable or diverging CN states (see Methods). The density and box plots here illustrate the mean arrival time of SCNAs that are shared (teal color) or diverging (salmon color) between-samples in each patient. The box represents the interquartile range, covering the central 50% of the data. The line inside the box indicates the median. Each pair of points linked with dashed line refers to a patient. Source data are provided as a Source Data file.

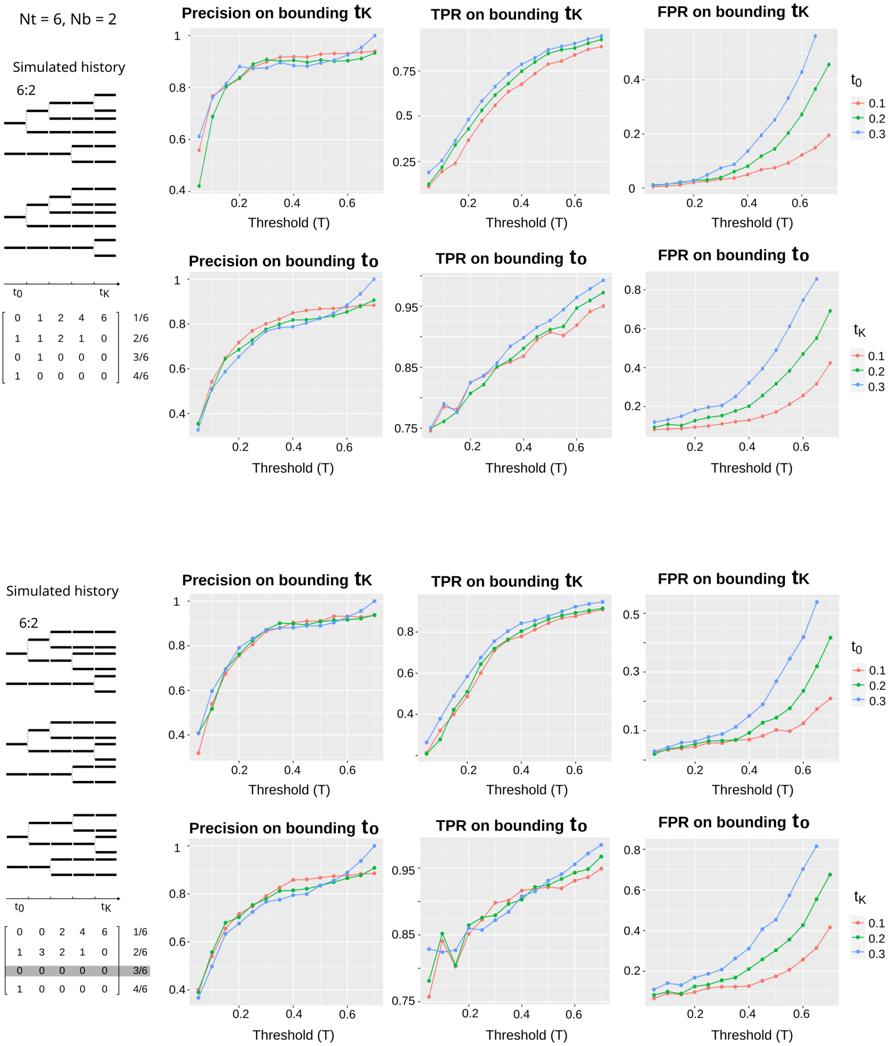

Supplementary Figure S 11 The figure illustrates `Butte`'s performance in identifying early and late gains using a predetermined timing threshold (T). The left panel displays two distinct histories for CN configuration 6:2. To assess late gains, a fixed t_0 (at 0.1, 0.2, or 0.3) was chosen, and SSNV data were simulated based on randomly generated t_K values. `Butte` was then applied, and the comparison between T and the predicted and actual timing values (detailed in the Methods section) allowed determination of true positive, false positive, true negative, and false negative outcomes. Similar procedures were employed to evaluate early gains. The right panel shows the precision, true positive rate (TPR), and false positive rate (FPR) for accurately bounding either t_0 or t_K. Source data are provided as a Source Data file.

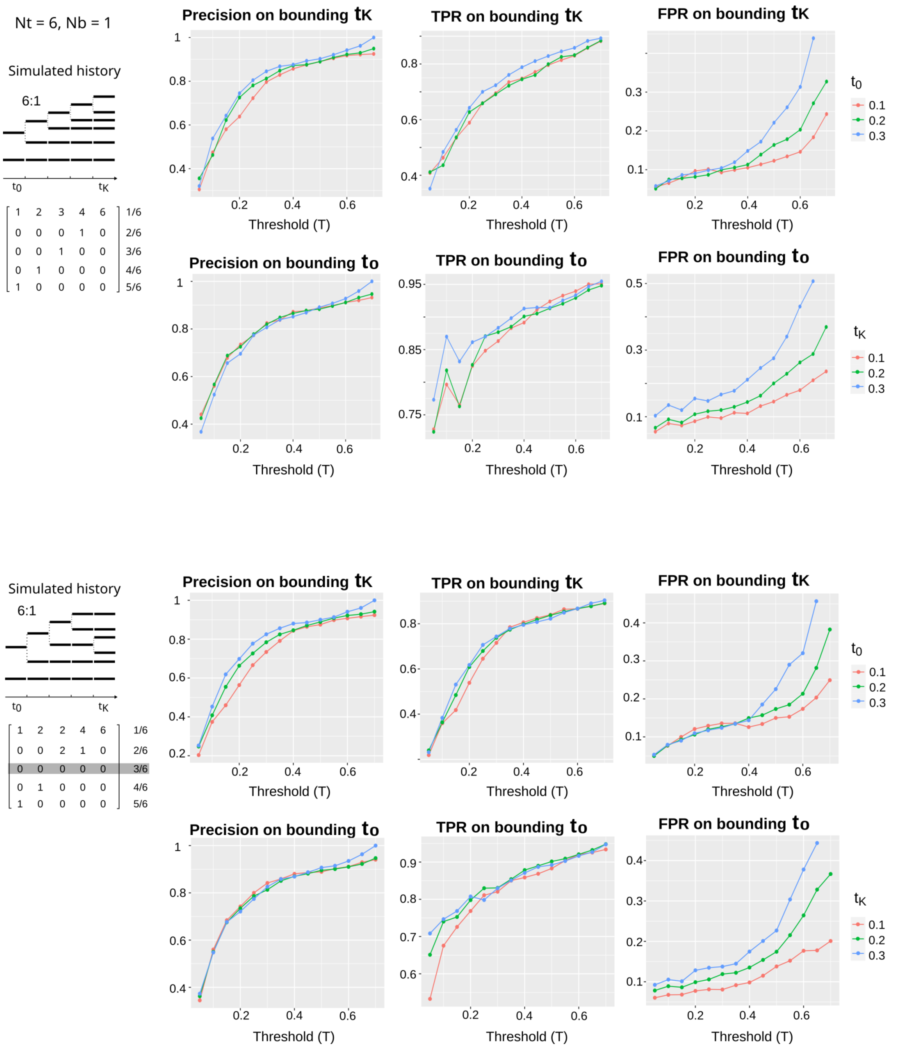

Supplementary Figure S 12 The figure illustrates `Butte`'s performance in identifying early and late gains using a predetermined timing threshold (T). The left panel displays two distinct histories for CN configuration 6:1. To assess late gains, a fixed t_0 (at 0.1, 0.2, or 0.3) was chosen, and SSNV data were simulated based on randomly generated t_K values. `Butte` was then applied, and the comparison between T and the predicted and actual timing values (detailed in the Methods section) allowed determination of true positive, false positive, true negative, and false negative outcomes. Similar procedures were employed to evaluate early gains. The right panel shows the precision, true positive rate (TPR), and false positive rate (FPR) for accurately bounding either t_0 or t_K. Source data are provided as a Source Data file.

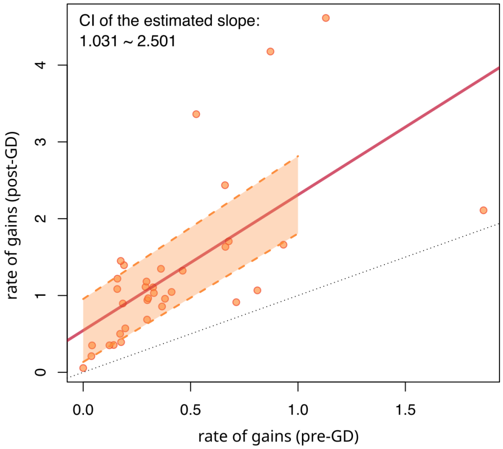

Supplementary Figure S 13 The rate of gains post-GD is compared against the rate pre-GD for each tumor. Only tumors with GD timed between 0.3 and 0.7 are included. The confidence interval of the estimated slope (or $\beta 1$) of the linear regression is indicated. The overall regression was statistically significant with $R^2 = 0.42$, F statistics = 23.9, degree of freedom = 33, and p value = 2.563e-05. Source data are provided as a Source Data file.

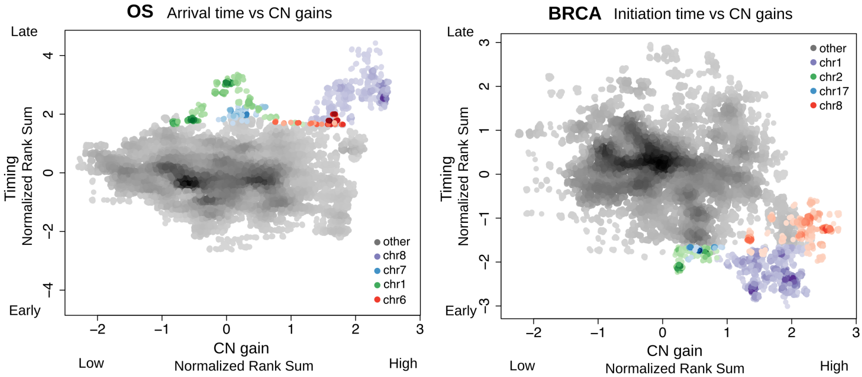

Supplementary Figure S 14 The figure illustrates the correlation between the CN gain and timing of gain in OS (n=38 tumor samples, arrival time) and BRCA (n=37 tumor samples, initiation time). Each point on the plots represents the timing of gain for a genomic bin plotted against its CN gain across patients. Colors denote specific chromosome regions, and color saturation reflects point density. The timings and CN gains were converted into normalized rank sums, as described in the Methods section. Source data are provided as a Source Data file.

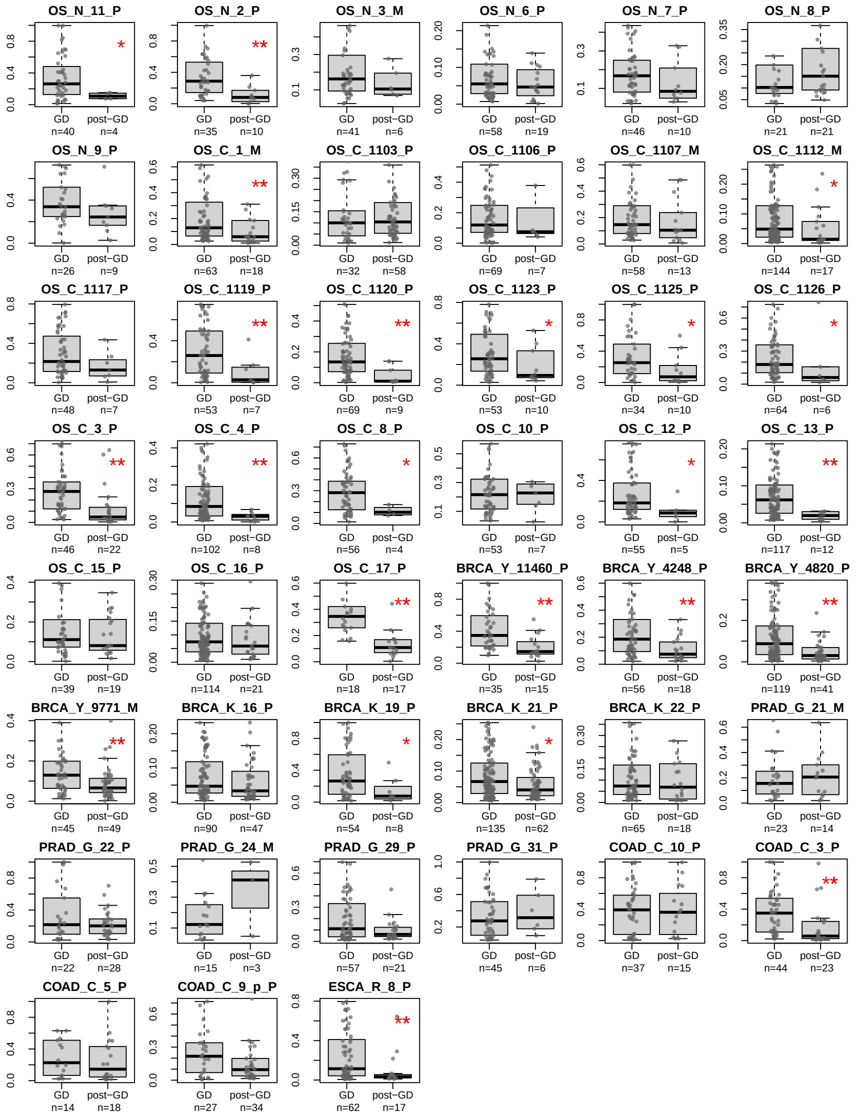

Supplementary Figure S 15 The box plots compare the segment length distributions between SCNAs occurred during GD and SCNAs post-GD. The segment length is normalized by the length of corresponding chromosome. Each panel refers to a patient tumor. Red stars indicate significance levels of Wilcoxon rank sum tests (one star: $p < 0.05$; two stars: $p < 0.01$). Sample sizes (n) are indicated for each category. The box represents the interquartile range, covering the central 50% of the data. The line inside the box indicates the median. Whiskers extend to the minimum and maximum values within a specified range, excluding outliers. Source data are provided as a Source Data file.

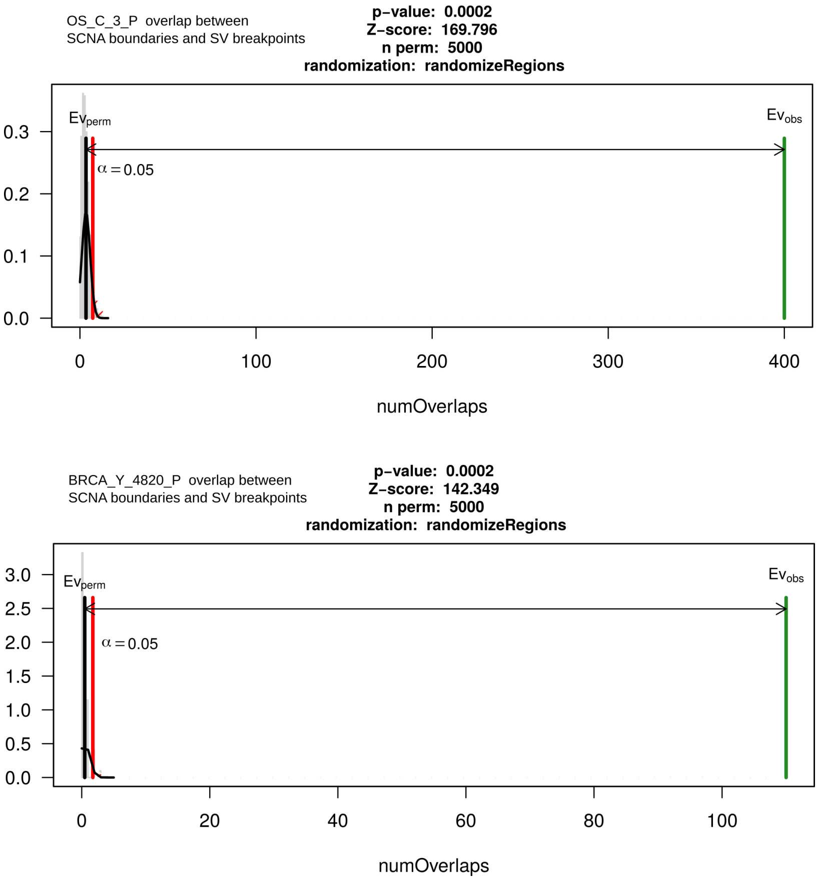

Supplementary Figure S 16 SV breakpoints locate at boundary regions of SCNA segments. The two plots visualize results of overlapPermTest function in R package `regioneR` [9], that statistically evaluates the number of overlaps between the SV breakpoints and boundary regions (±5000bp) of SCNAs in two samples. The green line shows the number of overlaps. On the other hand, the number of overlaps of the randomized genomic regions with SV breakpoints cluster around the black line. The red line denotes the significance limit. Source data are provided as a Source Data file.

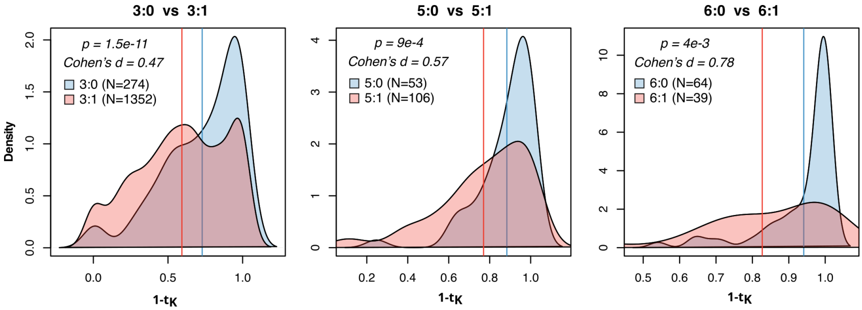

Supplementary Figure S 17 Comparisons of the arrival time between amplified loss-of-heterozygosity (ALOH) and allele-specific amplifications (ASA) at the same total CN. For a total CN $N_t >= 3$, ALOH has the minor allele CN $N_b = 0$ and ASA has $N_b = 1$. The density curves represent the distribution of the arrival time for ALOH (blue colored) and ASA (red) across the analyzed tumors, respectively. For N_t at 3, 5 and 6, ALOH established significantly later than ASA (as indicated by the p values, Wilcoxon rank sum test). Also shown is the Cohen's d as an estimate of the effect size. Vertical red and blue lines indicates the mean values for the two types of SCNAs, respectively. N: number of segments for the corresponding SCNA state. Source data are provided as a Source Data file.

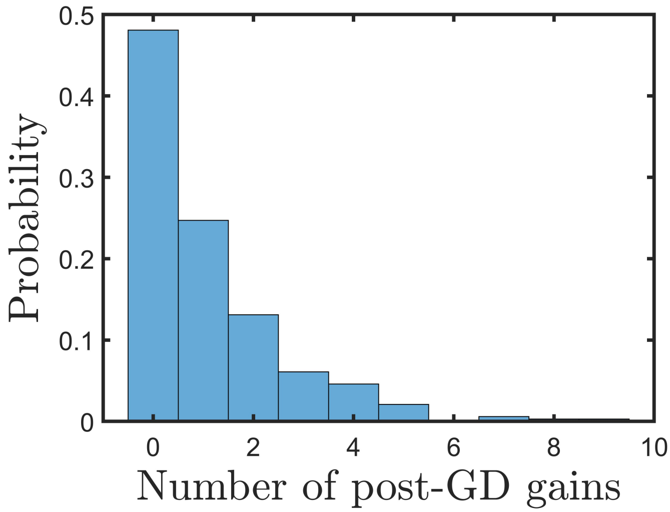

Supplementary Figure S 18 Histogram of number of dominant post-GD gains. Histogram is based on 10^3 simulations and all simulations used the model parameters: $a_0 = 1$, $b_0 = 0.5$, and $u_0 = 0.5$. Source data are provided as a Source Data file.

		Death rate of GD (b_0)								
		1	0.95	0.9	0.85	0.8	0.75	0.7	0.65	0.6
Birth rate of GD (a_0)	1.05	4.44	2.22	1.48	1.11	0.88	0.74	0.63	0.55	0.49
	1.10	2.22	1.48	1.11	0.88	0.74	0.63	0.55	0.49	0.44
	1.15	1.48	1.11	0.88	0.74	0.6	0.55	0.49	0.44	0.40
	1.20	1.11	0.88	0.74	0.63	0.55	0.49	0.44	0.40	0.37
	1.25	0.88	0.74	0.63	0.55	0.49	0.44	0.40	0.37	0.34
	1.30	0.74	0.63	0.55	0.49	0.44	0.40	0.37	0.34	0.31
	1.35	0.63	0.55	0.49	0.44	0.40	0.37	0.34	0.31	0.29
	1.40	0.55	0.49	0.44	0.40	0.37	0.34	0.31	0.29	0.27
	1.45	0.49	0.44	0.40	0.37	0.34	0.31	0.29	0.27	0.26

		Rate of beneficial post-GD gains (u_1)								
		3e-5	5e-5	7e-5	9e-5	1.1e-4	1.3e-4	1.5e-4	1.7e-4	1.9e-4
Birth rate of GD (a_0)	1.05	22.30	20.24	18.89	17.88	17.08	16.41	15.84	15.34	14.90
	1.10	14.06	13.03	12.36	11.86	11.45	11.12	10.83	10.58	10.36
	1.15	10.54	9.86	9.41	9.08	8.81	8.58	8.39	8.23	8.08
	1.20	8.5	8.04	7.70	7.45	7.25	7.08	6.94	6.81	6.70
	1.25	7.25	6.84	6.57	6.37	6.21	6.08	5.96	5.86	5.78
	1.30	6.33	5.99	5.77	5.60	5.47	5.36	5.26	5.18	5.10
	1.35	5.65	5.35	5.16	5.02	4.90	4.81	4.73	4.66	4.59
	1.40	5.11	4.86	4.69	4.56	4.46	4.38	4.31	4.24	4.19
	1.45	4.68	4.45	4.30	4.19	4.10	4.03	3.97	3.91	3.86

Supplementary Figure S 19 The top table: the expected number of post-GD gains of model 1 with respect to different birth rates (a_0) and death rates of GD (b_0). Other parameters for the top table: $u_0 = 0.2$. The bottom table: the expected number of post-GD gains of model 2 with respect to different birth rates of GD (a_0) and rates of beneficial post-GD gains (u_1). Other parameters for the bottom table: $b_0 = 1$, $a_1 = 1.5$, $b_1 = 1$, and $u_0 = 0.2$.

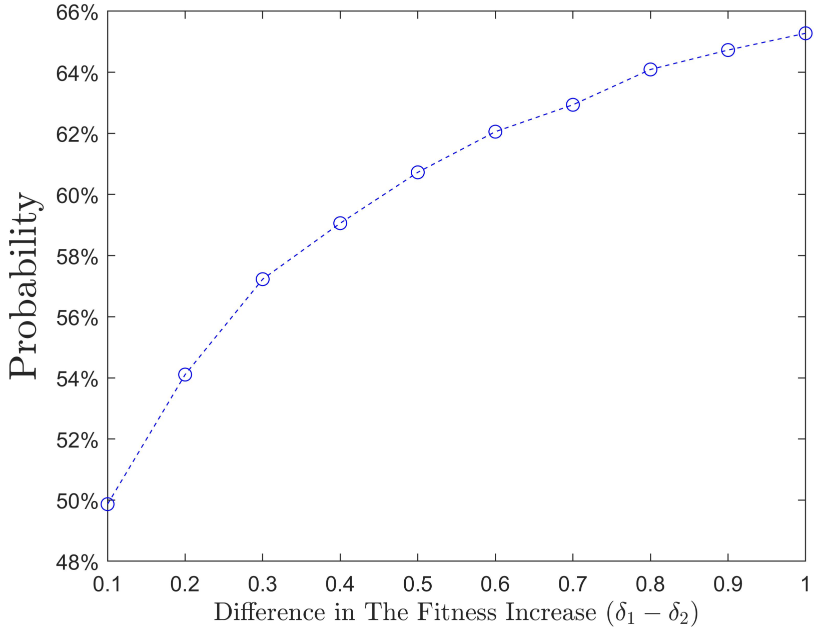

Supplementary Figure S 20 The probability of the first dual-mutation cell with mutation one occurring first. This plot displays the simulation data of a two driver mutation model, where mutation one results in an increase of fitness of δ_1 and mutation two results in an increase of fitness of $\delta_2 < \delta_1$. We simulate the tumor from a single cell without any mutation until the first cell with both mutations that does not go extinct. The parameters are as follows: birth rate for cells without any mutation $a_0 = 1$, death rate for cells without any mutation $b_0 = 1$, birth rate for cells with mutation one only $a_1 = 1 + \delta_1$, death rate for cells with mutation one only $b_1 = b_0$, birth rate for cells with mutation two only $a_2 = 1 + \delta_2 = 1.1$, death rate for cells with mutation two only $b_2 = b_0$, birth rate for cells with both mutations $a_3 = 1 + \delta_1 + \delta_2$, death rate for cells with both mutations $b_3 = b_0$, mutation rate for mutation one $u_1 = 0.2$, mutation rate for mutation two $u_2 = 0.2$. Each point is computed from 100,000 simulations. Source data are provided as a Source Data file.

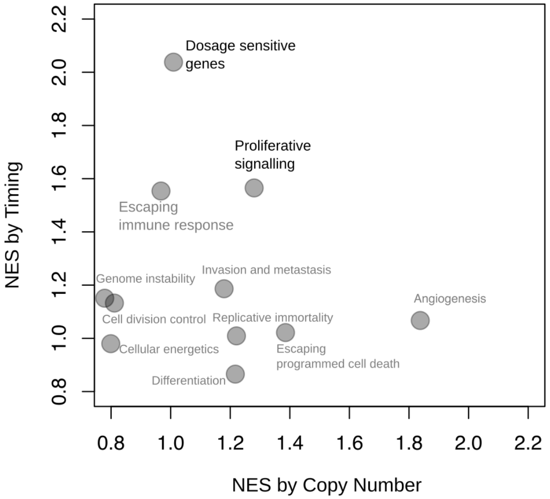

Supplementary Figure S 21 The comparison of gene set enrichment analysis (GSEA) results between the gene ranks by averaged timing (as shown in Figure 6A) and the ranks by their copy number (CN) in the OS cohort. Gene ranking by copy number was performed using normalized rank sums derived from log2 copy number ratio data. This process involved converting segment mean values of log2 copy number ratios into ranks within each sample. Subsequently, the middle rank of each respective sample was subtracted from individual gene ranks, and these adjusted ranks were summed across patients for each gene. Normalized rank sums were obtained by dividing the rank sums by their standard deviation. The scatter plot illustrates the Normalized Enrichment Scores (NES) for cancer census genes categorized under predefined cancer hallmarks according to the COSMIC database. Source data are provided as a Source Data file.

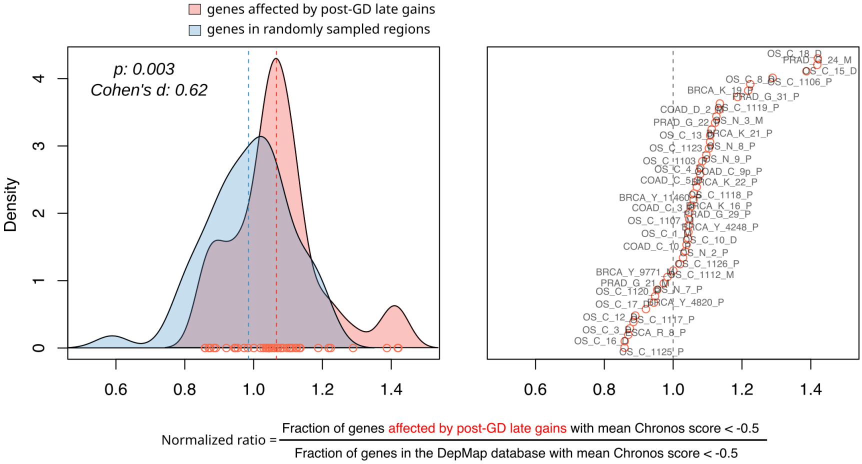

Supplementary Figure S 22 Evaluating the fitness effect of post-GD late appearing gains using the gene based Chronos score [10] provided in the DepMap database (https://depmap.org/portal/). The Chronos score reflects the change in cell proliferation upon the Cripsr knockout of the respective gene in a particular cell line. A lower negative Chronos score indicates that the gene has a positive effect on maintaining cell survival and division. For simplicity, we took the average score for each gene across all the cell lines. For each tumor, we calculated the fraction of genes affected by late gains with a mean Chronos score < -0.5. We then obtained the normalized ratio (**NR**) by dividing it by the ratio calculated from all the genes in the database. An NR of 1 (black dotted line on the right panel) means that the genes of interest are not different from a genome-wide background in terms of the knock-out fitness effect. Interestingly, we found that most patient tumors have an NR greater than 1 (the right panel), so does the mean NR across patients (left panel, red dotted line). By contrast, genes from randomly sampled genomic regions have a significantly smaller NR ($p = 0.003$, Wilcoxon rank sum test; effect size $= 0.62$) with the mean close to 1 (left panel, blue dotted line). The randomized regions keep the same number and length of segments as the late gains in each patient (by using function randomizeRegions in R package `regioneR` [9]). Source data are provided as a Source Data file.

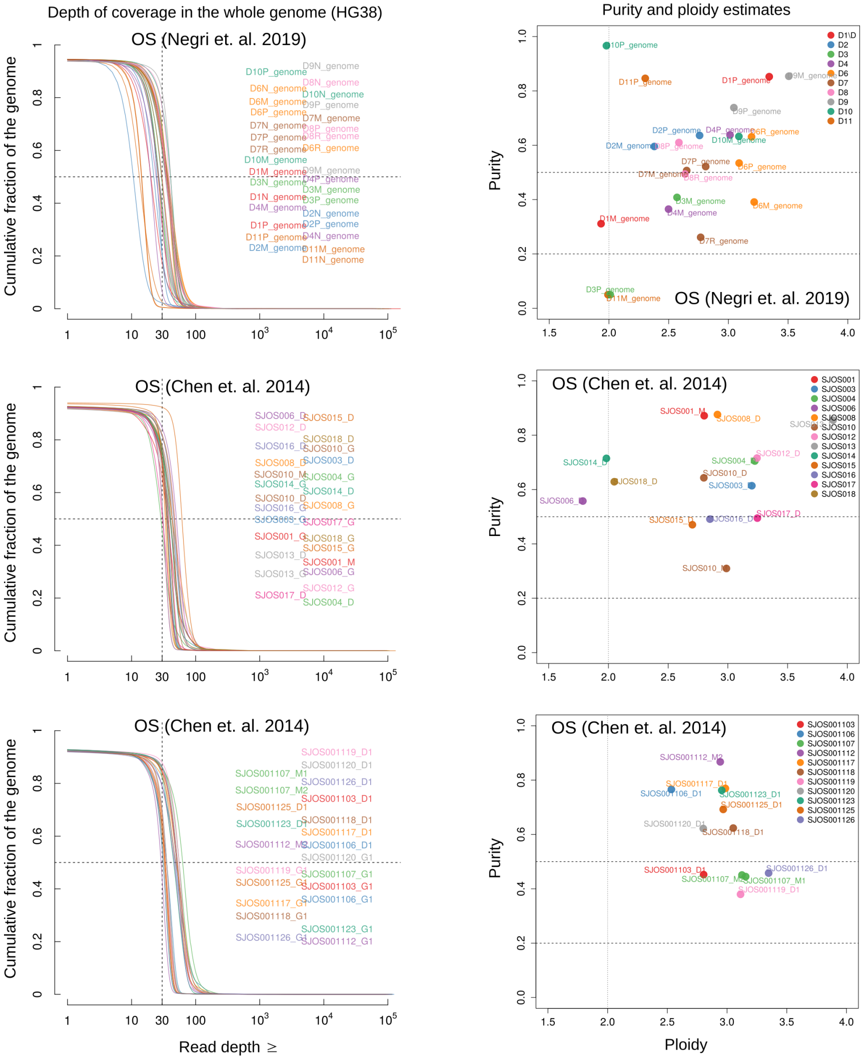

Supplementary Figure S 23 Quality control assessment of OS WGS datasets. Two independent OS datasets [11, 12] were evaluated. For each dataset, the cumulative faction of the human genome (hg38) covered at or above a given read depth is shown on the left panel. Sample IDs are labelled and sorted according to the depth of coverage (i.e., fraction of genome covered at a minimum depth of 30) in the decreasing order. The right panel shows tumor purity relative to ploidy, where both values were estimated based on TitanCNA [13]. Samples with estimated purity less than 0.2 were excluded for the analysis. Source data are provided as a Source Data file.

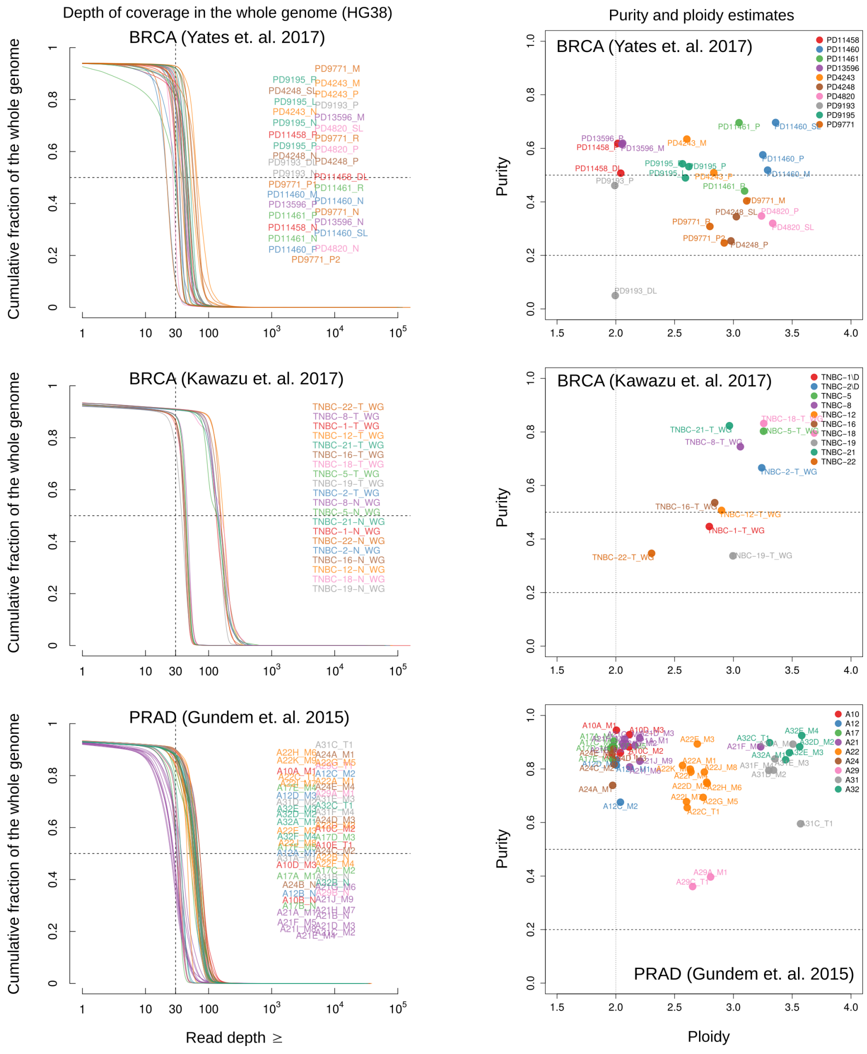

Supplementary Figure S 24 Quality control assessment of BRCA and PRAD WGS datasets. Two independent BRCA datasets [14, 15] and a PRAD [16] dataset were evaluated. For each dataset, the cumulative faction of the human genome (hg38) covered at or above a given read depth is shown on the left panel. Sample IDs are labelled and sorted according to the depth of coverage (i.e., fraction of genome covered at a minimum depth of 30) in the decreasing order. The right panel shows tumor purity relative to ploidy, where both values were estimated based on TitanCNA [13]. Source data are provided as a Source Data file.

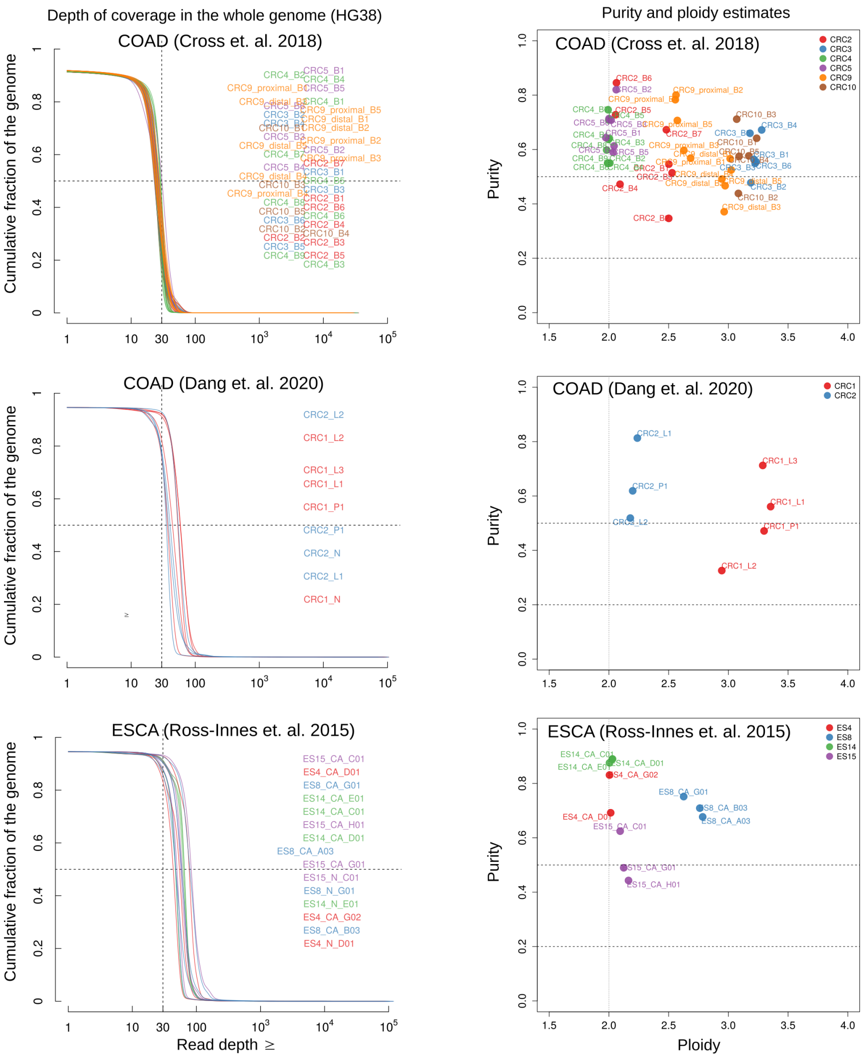

Supplementary Figure S 25 Quality control assessment of COAD and ESCA WGS datasets. Two independent COAD datasets [8, 17] and an ESCA [18] dataset were evaluated. For each dataset, the cumulative faction of the human genome (hg38) covered at or above a given read depth is shown on the left panel. Sample IDs are labelled and sorted according to the depth of coverage (i.e., fraction of genome covered at a minimum depth of 30) in the decreasing order. The right panel shows tumor purity relative to ploidy, where both values were estimated based on TitanCNA [13]. Source data are provided as a Source Data file.

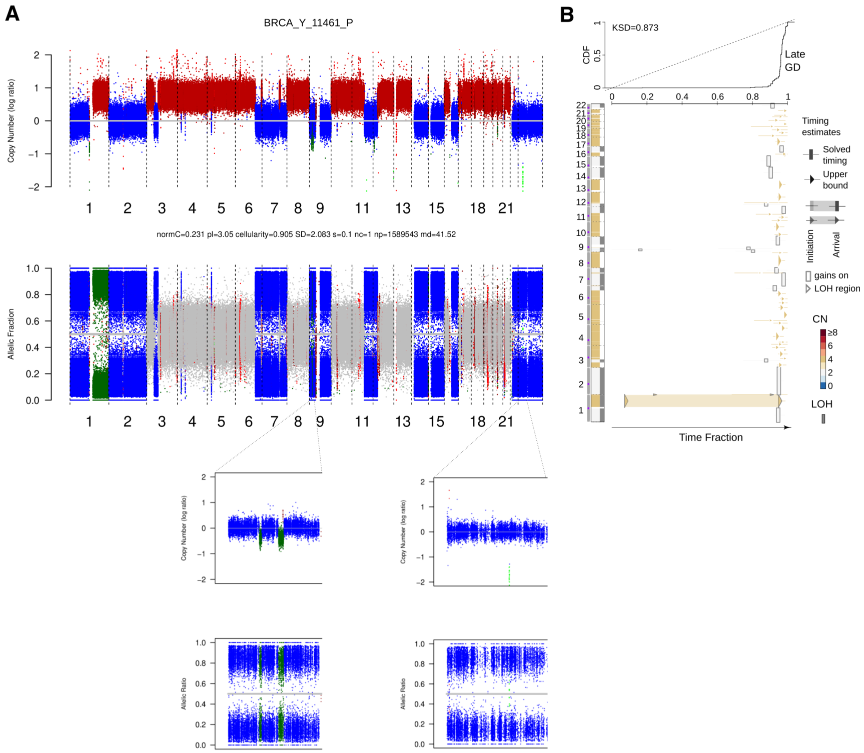

Supplementary Figure S 26 For copy number calling, the ploidy baseline (CN = 2) is determined by the model complexity in explaining the log read ratio and allelic imbalance of heterozygous SNPs in the corresponding patient (conventionally referred to as logR and BAF, respectively). (**A**) For example, for sample BRCA_Y_11461_P, if we set the baseline to the log ratio of the red-colored segments (e.g., chr4), half of the genome (i.e., the blue-colored segments) would be at haploid state with only one copy. This would cause problems during mitosis which limit the viability of the cell. Moreover, one has to introduce a subclone to explain the green segments on chr9 with lower logR and similar allelic imbalance as compared to the blue segments. Therefore, we established the baseline at the blue segments, which leads to a simpler one-clone model to explain the profiles holistically across chromosomes, including the deletions on chr9 and the focal homozygous deletion on chrX. (**B**) The timing result of BRCA_Y_11461_P, where the genome doubling appears to be quite close to the MRCA. Source data are provided as a Source Data file.

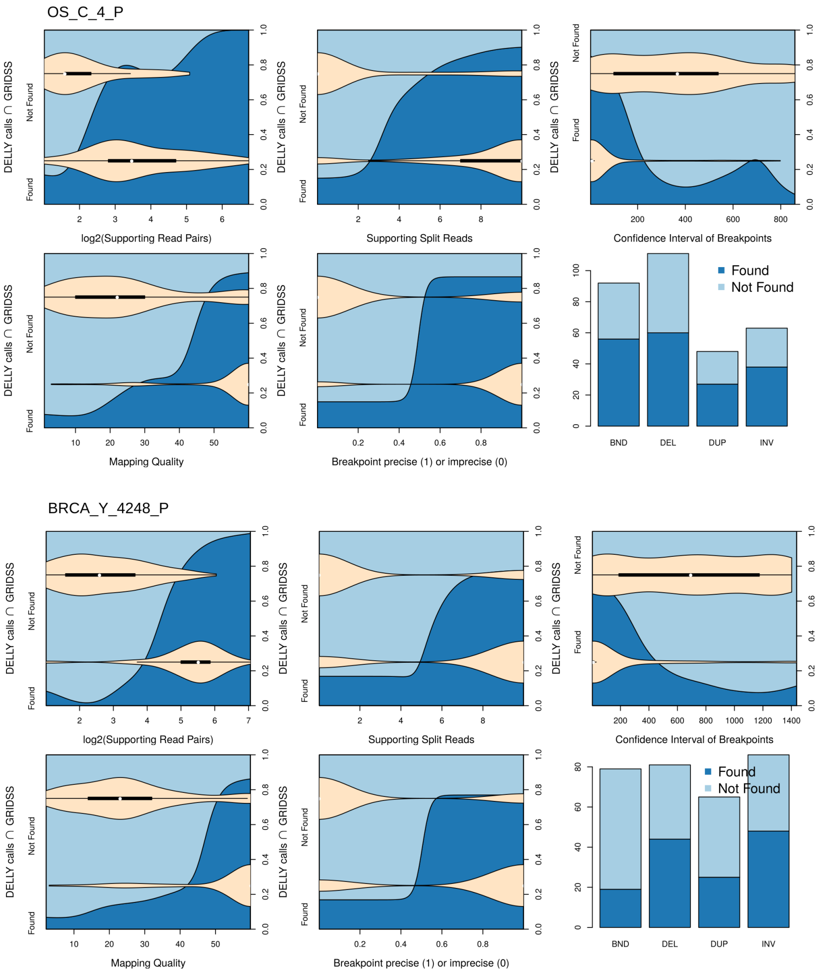

Supplementary Figure S 27 Conditional density plots compare the quality metrics of SV calls shared by DELLY [19] and GRIDSS [20] (labeled as Found) and SV calls unique to DELLY (Not Found). Two example patient tumors are shown. The supporting evidence are much stronger for calls that are found by both tools. Source data are provided as a Source Data file.

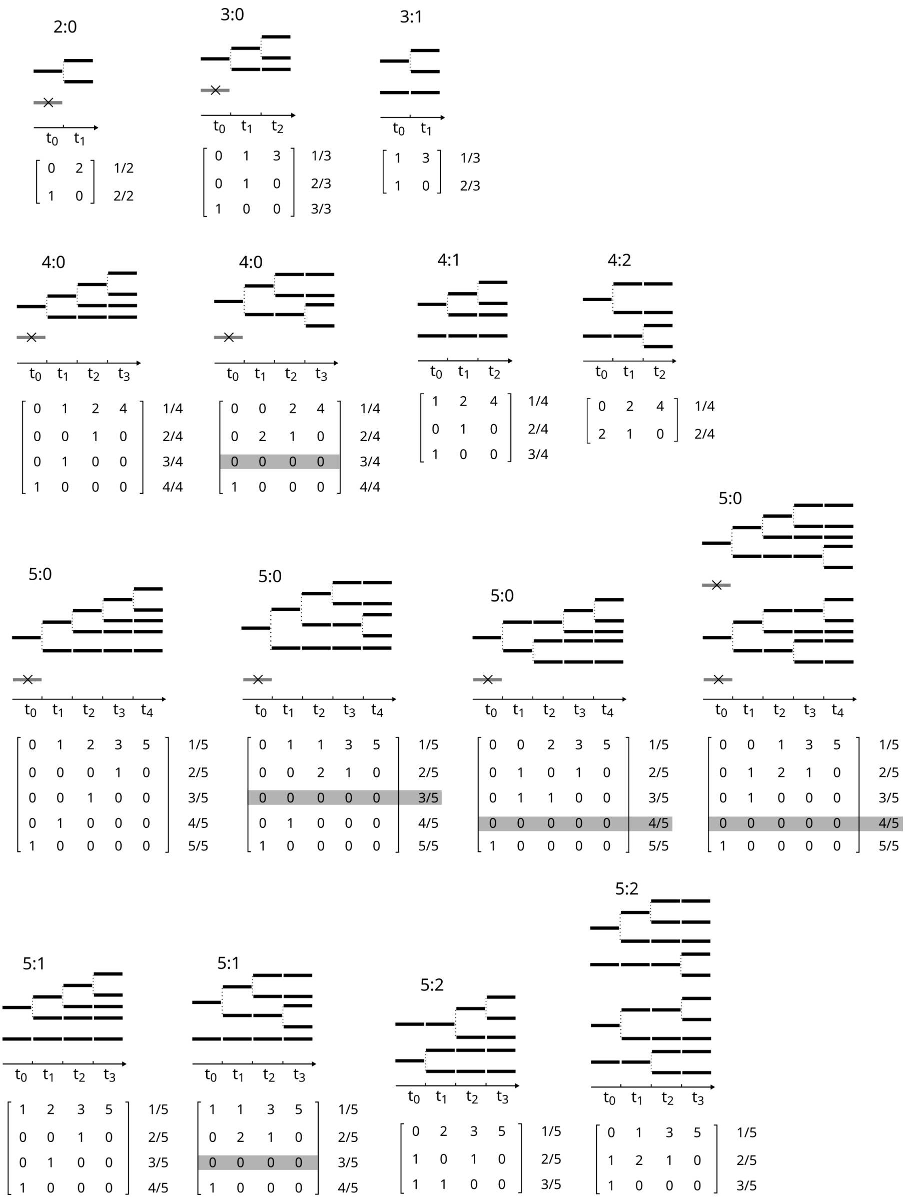

Supplementary Figure S 28 History matrices for SCNA with a total CN less than 6.

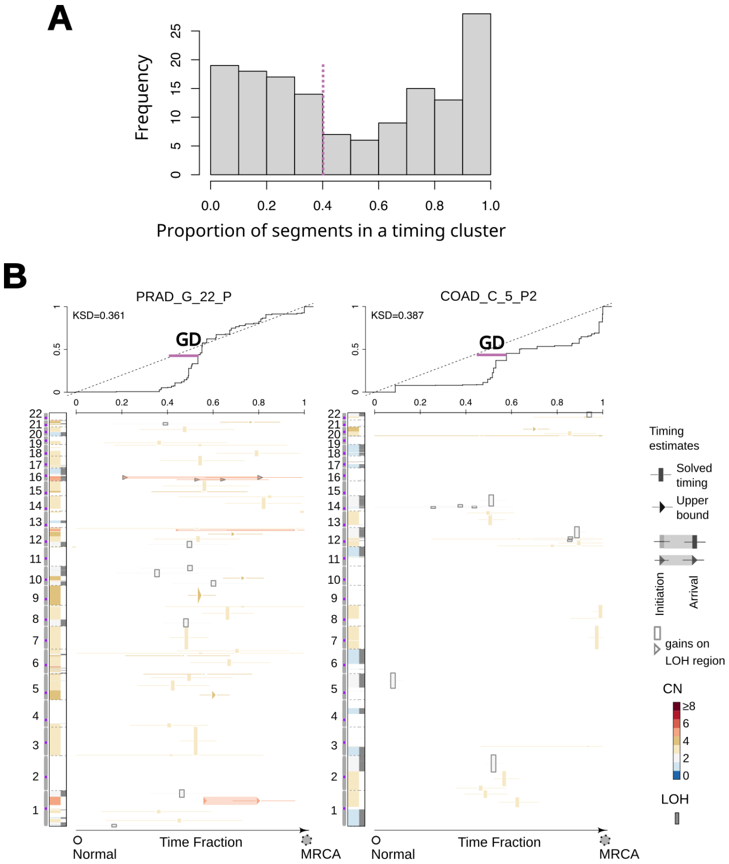

Supplementary Figure S 29 (A) The histogram displays the proportion of segments in timing clusters. (B) Two sample tumors where GD is identified as the timing cluster encompassing over 40% of SCNA segments. Source data are provided as a Source Data file.

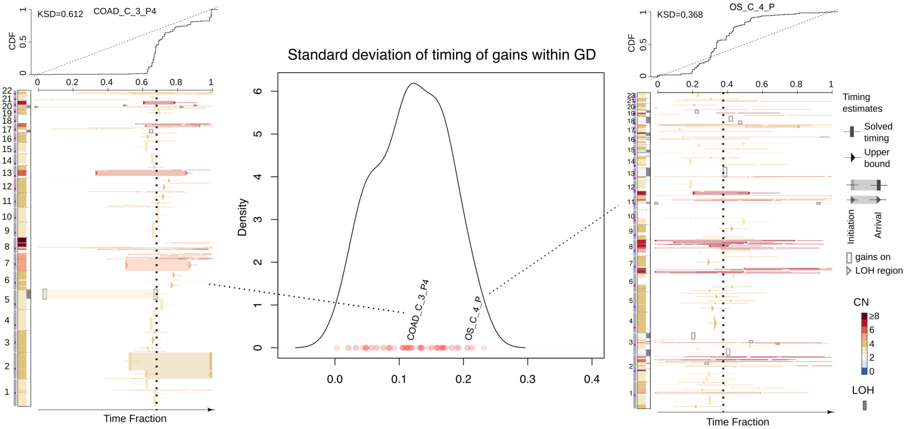

Supplementary Figure S 30 The timing of gains within a GD is tightly distributed. The middle panel displays the density of standard deviation of the timing within GD across 67 patients (shown as red dots). The left panel shows the SCNA timing for a patient tumor (COAD_C_3_P4) with the standard deviation at 0.13. The right panel shows another tumor (OS_C_4_P) with a more dispersed timing patterns within GD (the standard deviation is greater than 0.2). For rare cases such as OS_C_4_P, it is possible that the genome reached the high ploidy state via sequential acquisition of gains, instead of a single event. Source data are provided as a Source Data file.

Supplementary References

[1] Gunnarsson, E. B., Leder, K. & Foo, J. Exact site frequency spectra of neutrally evolving tumors: a transition between power laws reveals a signature of cell viability. *Theoretical Population Biology* (2021). URL https://arxiv.org/abs/2102.11959v2.

[2] Durrett, R. Branching Process Models of Cancer. *Branching Process Models of Cancer. Mathematical Biosciences Institute Lecture Series* **1.1**, 1–63 (2015).

[3] O'Connell, N. Yule process approximation for the skeleton of a branching process. *Journal of Applied Probability* **30**, 725–729 (1993).

[4] Alexander, H. K. Conditional distributions and waiting times in multitype branching processes. *Advances in Applied Probability* **45**, 692–718 (2013). URL https://projecteuclid.org/journals/advances-in-applied-probability/volume-45/issue-3/Conditional-distributions-and-waiting-times-in-multitype-branching-processes/10.1239/aap/1377868535.fullhttps://projecteuclid.org/journals/advances-in-applied-probabilit.

[5] Nik-Zainal, S. *et al.* The life history of 21 breast cancers. *Cell* **149**, 994–1007 (2012). URL citeulike-article-id:10679933http://dx.doi.org/10.1016/j.cell.2012.04.023.

[6] Greenman, C. D. *et al.* Estimation of rearrangement phylogeny for cancer genomes. *Genome Research* **22**, 346–361 (2012).

[7] Campbell, P. J. *et al.* Pan-cancer analysis of whole genomes. *Nature* **578**, 82–93 (2020). URL https://doi.org/10.1038/s41586-020-1969-6.

[8] Cross, W. *et al.* The evolutionary landscape of colorectal tumorigenesis. *Nature Ecology & Evolution* **2**, 1661–1672 (2018). URL http://www.nature.com/articles/s41559-018-0642-z.

[9] Gel, B. *et al.* RegioneR: An R/Bioconductor package for the association analysis of genomic regions based on permutation tests. *Bioinformatics* **32**, 289–291 (2015). URL http://www.ncbi.nlm.nih.gov/pubmed/26424858http://www.pubmedcentral.nih.gov/articlerender.fcgi?artid=PMC4708104https://academic.oup.com/bioinformatics/article-lookup/doi/10.1093/bioinformatics/btv562.

[10] Dempster, J. M. *et al.* Chronos: a cell population dynamics model of CRISPR experiments that improves inference of gene fitness effects. *Genome Biology* **22**, 1–23 (2021). URL https://genomebiology.biomedcentral.com/articles/10.1186/s13059-021-02540-7.

[11] Negri, G. L. *et al.* Integrative genomic analysis of matched primary and metastatic pediatric osteosarcoma. *The Journal of Pathology* **249**, 319–331 (2019). URL https://onlinelibrary.wiley.com/doi/abs/10.1002/path.5319.

[12] Chen, X. *et al.* Recurrent Somatic Structural Variations Contribute to Tumorigenesis in Pediatric Osteosarcoma. *Cell Reports* **7**, 104–112 (2014). URL http://www.ncbi.nlm.nih.gov/pubmed/24703847http://www.pubmedcentral.nih.gov/articlerender.fcgi?artid=PMC4096827http://linkinghub.elsevier.com/retrieve/pii/S221112471400165X.

[13] Ha, G. *et al.* TITAN: Inference of copy number architectures in clonal cell populations from tumor whole-genome sequence data. *Genome Research* **24**, 1881–1893 (2014). URL http://genome.cshlp.org/content/24/11/1881.full.

[14] Yates, L. R. *et al.* Genomic Evolution of Breast Cancer Metastasis and Relapse. *Cancer Cell* **32**, 169–184.e7 (2017). URL http://dx.doi.org/10.1016/j.ccell.2017.07.005.

[15] Kawazu, M. *et al.* Integrative analysis of genomic alterations in triple-negative breast cancer in association with homologous recombination deficiency. *PLOS Genetics* **13**, e1006853 (2017). URL http://dx.plos.org/10.1371/journal.pgen.1006853.

[16] Gundem, G. *et al.* The evolutionary history of lethal metastatic prostate cancer. *Nature* **520**, 353–357 (2015). URL http://www.nature.com/doifinder/10.1038/nature14347.

[17] Dang, H. X. *et al.* The clonal evolution of metastatic colorectal cancer. *Science Advances* **6**, eaay9691 (2020). URL https://advances.sciencemag.org/lookup/doi/10.1126/sciadv.aay9691.

[18] Ross-Innes, C. S. *et al.* Whole-genome sequencing provides new insights into the clonal architecture of Barrett's esophagus and esophageal adenocarcinoma. *Nature Genetics* **47**, 1038–1046 (2015). URL http://www.nature.com/doifinder/10.1038/ng.3357.

[19] Rausch, T. *et al.* DELLY: structural variant discovery by integrated paired-end and split-read analysis. *Bioinformatics* **28**, i333–i339 (2012). URL http://www.ncbi.nlm.nih.gov/pubmed/22962449http://www.pubmedcentral.nih.gov/articlerender.fcgi?artid=PMC3436805.

[20] Cameron, D. L. *et al.* GRIDSS: Sensitive and specific genomic rearrangement detection using positional de Bruijn graph assembly. *Genome Research* **27**, 2050–2060 (2017).
